# Donation interview before to brain death

**DOI:** 10.1186/2197-425X-3-S1-A903

**Published:** 2015-10-01

**Authors:** A Fernandez Carmona, M Sevilla Martínez, JM Perez Villares, R Lara Rosales, EP Fuentes Garcia

**Affiliations:** Virgen de las Nieves Hospital, Intensive Care Medicine, Granada, Spain; Hospital Universitario San Cecilio, Intensive Care Medicine, Granada, Spain

## Introduction

The family interview to request donation is one of the key stages of obtaining organs for transplantation. The donation interview before to brain death (DIBBD) is done in two stages: in the emergency room or hospital ward, with prior notification of the existence of a severe neurological injury with a fatal short-term prognosis and which is dismissed surgery or ICU admission or when the family members of patients are informed of the seriousness of the process and requesting the withdrawal of life support measures.

## Objectives

Analyze the results of DIBBD performed in a single tertiary level centre.

## Material and Methods

Unicentric longitudinal descriptive study. Including all DIBBD performed since January 2011 until October 2014. All cases were registered in a focused database, including demographics data, place where the interview take place, time from admission to interview, time from interview to death and interview result and donation data (real and effective donors).

## Results

During this period, 38 DIBBD were performed. The DIBBD during this period was 34% of donation interviews realized in our center.

Family approval to organ donation in case of brain death was 92,10% (35).

60,5% of DIBBD were performed in a emergency room. The reasons for emergency admission were: hemorrhagic stroke in 74% of cases, ischemic stroke 13% and Traumatic Brain 3%, others 10%.

The mean age average of patients admitted to the ICU after DIBBD was 73,5 years.

Within the group of patients admitted in ICU after DIBBD, the donation was contraindicated in 5 patients, also 5 patients died from cardiac arrest. All of the rest of the series developed encephalic death (25 patients) and 80% were effective donors (20 patients).

## Conclusions

DIBBD results was very satisfactory, the results was positive in 92,10% of cases.

In our centre DIBBD implementation has increased potential donors detection as well as the final number of donations. Improving others professionals implication (emergency area, hospital ward…) in this process.

Most of the DIBBD was realized to families of 70-year-old major patients.

